# The effects of different exercise interventions on clinical outcomes of irritable bowel syndrome and their potential mechanisms: a systematic review and network meta-analysis

**DOI:** 10.3389/fimmu.2026.1730624

**Published:** 2026-02-11

**Authors:** Zheping Quan, Weijia Song, Qianting Huang, Jiale Wang

**Affiliations:** 1 Department of Physical Education, Taiyuan Normal University, Jinzhong, Shanxi, China; 2 College of Sports Physical Education and Recreation, Cavite State University, Cavite, Philippines; 3 Physical Education and Sports, Belarusian National Technical University, Minsk, Belarus

**Keywords:** exercise intervention, gut-immune axis, irritable bowel syndrome, network meta-analysis, systematic review

## Abstract

**Background:**

Irritable bowel syndrome (IBS) is a functional gastrointestinal disorder characterized by recurrent abdominal pain or discomfort accompanied by altered bowel habits, often alongside psychological symptoms. Increasing evidence suggests that exercise interventions not only alleviate clinical symptoms but may also exert effects through modulation of the gut-immune axis. This study aims to systematically compare the effects of different exercise modalities on symptoms, quality of life, and psychological status in IBS patients, whilst exploring potential immunological mechanisms.

**Methods:**

The PubMed, Cochrane Library, EMBASE, and Web of Science databases were systematically searched for literature from inception to June 2025, and conventional Meta-analysis and Web Meta-analysis were performed using RevMan 5.4 and R 4.3.3 software.

**Results:**

Sixteen studies were included, covering interventions such as running, moderate-intensity aerobic exercise, Pilates, yoga, and Baguazhang. Moderate-intensity aerobic exercise and Pilates demonstrated significant effects (P < 0.05) in improving depression and anxiety scores, as well as IBS-SSS and IBS-QOL scores. Running and aerobic exercise showed the best overall efficacy. Mechanistically, exercise interventions may have a synergistic effect on the brain-gut-immune axis by enhancing parasympathetic activity, modulating the HPA axis, improving gut microbiota, strengthening intestinal barrier function, and reducing systemic inflammation. This approach alleviates gastrointestinal symptoms while enhancing mental wellbeing.

**Conclusion:**

Various exercise interventions may improve clinical symptoms and quality of life in patients with irritable bowel syndrome to a certain extent, and may possess potential immunomodulatory effects. As a relatively safe and cost-effective non-pharmacological treatment, exercise holds considerable clinical application potential in the comprehensive management of irritable bowel syndrome.

**Systematic Review Registration:**

https://www.crd.york.ac.uk/PROSPERO/, identifier CRD420251132835.

## 1 Introduction

Irritable bowel syndrome is a functional gastrointestinal disorder characterized by abdominal pain and altered bowel habits (1, 2), frequently co-occurring with psychological comorbidities such as anxiety and depression. Its pathophysiological core lies in dysfunction of the brain-gut axis (3, 4). Beyond neuropsychological mechanisms, immune dysregulation—including low-grade inflammation, intestinal barrier dysfunction, and microbiota disturbance—constitutes a pivotal component of IBS pathophysiology. This immune component also serves as a bridge linking intestinal symptoms to systemic health (5, 6).

Exercise intervention, as an effective non-pharmacological approach, not only alleviates symptoms but may also exert effects by modulating the intestinal immune microenvironment (7). Its known physiological effects—such as anti-inflammatory action, regulation of stress systems, and improvement of gut environment—suggest potential therapeutic mechanisms via the brain-gut-immune axis (4). However, the differential efficacy of various exercise modalities in this regard remains unclear. Currently, while numerous randomized controlled trials (RCTs) have compared the effects of single exercise interventions with standard care, there remains a lack of systematic ranking and comparative analysis of the efficacy of different exercise modalities. More critically, comprehensive exploration at the systematic review level is lacking regarding the underlying mechanisms behind these efficacy differences, particularly those related to immunomodulation.

Therefore, this study aims to employ a network meta-analysis to systematically compare the relative efficacy of different exercise interventions on clinical symptoms and quality of life in patients with irritable bowel syndrome (IBS). Building upon this, by synthesizing the mechanistic descriptions within the existing evidence, we will explore the potential immunomodulatory pathways involved in different exercise interventions (such as effects on inflammation, the intestinal barrier, and the microbiota). This will provide a deeper biological perspective for understanding the differences in their efficacy and guide future research focusing on immune endpoints (8, 9).

## 2 Materials and methods

### 2.1 Registration

This study adheres to the Preferred Reporting Items for Systematic Reviews and Meta-analyses (PRISMA) guidelines. The research protocol has been registered on the International Prospective Systematic Reviews Register (PROSPERO) platform, with registration number CRD420251132835 (10).

### 2.2 Search strategy

The system retrieved relevant literature from four English-language databases: PubMed, Cochrane Library, EMBASE, and Web of Science. Articles were included from the inception of each database up to June 2025. The following MeSH terms were applied to search for relevant literature (the search strategy is illustrated using PubMed as an example):(“irritable bowel syndrome”[MeSH Terms] OR (“irritable”[All Fields] AND “bowel”[All Fields] AND “syndrome”[All Fields]) OR “irritable bowel syndrome”[All Fields]) AND (“exercise”[MeSH Terms] OR “exercise”[All Fields] OR “exercises”[All Fields] OR “exercise therapy”[MeSH Terms] OR (“exercise”[All Fields] AND “therapy”[All Fields]) OR “exercise therapy”[All Fields] OR “exercising”[All Fields] OR “exercise s”[All Fields] OR “exercised”[All Fields] OR “exerciser”[All Fields] OR “exercisers”[All Fields] OR (“sport s”[All Fields] OR “sports”[MeSH Terms] OR “sports”[All Fields] OR “sport”[All Fields] OR “sporting”[All Fields]) OR (“swimming”[MeSH Terms] OR “swimming”[All Fields] OR “swims”[All Fields]) OR (“yoga”[MeSH Terms] OR “yoga”[All Fields]) OR (“walked”[All Fields] OR “walking”[MeSH Terms] OR “walking”[All Fields] OR “walks”[All Fields]) OR (“jogged”[All Fields] OR “jogging”[MeSH Terms] OR “jogging”[All Fields]) OR (“tai ji”[MeSH Terms] OR (“tai”[All Fields] AND “ji”[All Fields]) OR “tai ji”[All Fields])).

### 2.3 Selection criteria

#### 2.3.1 Inclusion criteria

Studies included in the meta-analysis must meet all of the following inclusion criteria based on the PICOS principles (10): 1) Population: Patients clinically diagnosed with diarrhea-predominant, constipation-predominant, or mixed-type irritable bowel syndrome (IBS), with organic diseases ruled out via endoscopy, X-ray, ultrasound, and laboratory examinations; 2) Intervention: Intervention group receiving exercise intervention added to standard care or exercise intervention alone, including but not limited to yoga, running, Pilates, Baduanjin, and moderate-intensity aerobic exercise; 3) Comparison intervention: Control group receiving standard care alone; 4) Outcome measures: Report at least one of the following outcome measures: IBS-SSS scale scores, IBS-SSS subscale scores for Pain severity, Pain duration, Abdominal distension, Bowel satisfaction, Interference with life; IBS-QOL scale scores, IBS-QOL subscale scores for Dysphoria, Interference with activity, Body image, Health worries, Food avoidance, Social reaction, Sexual, Relationships; Depression scores, Anxiety scores; 5) Study design: clinically relevant RCT trials.

#### 2.3.2 Exclusion criteria

Exclusion criteria: 1) Presence of other gastrointestinal diseases besides irritable bowel syndrome(such as gastric ulcers, Crohn’s disease, ulcerative colitis, etc.); 2) Articles lacking assessment of the aforementioned outcome measures or failing to establish intergroup comparisons; 3) Failure to specify assessment time points; 4) Duplicate publications; 5) Combination with external therapies; 6) Inability to extract accurate or complete original data.

### 2.4 Data extraction

In the included studies, all original literature was incorporated into the EndNote X9 (11) academic management platform, and duplicate records were removed. Two researchers (WS and ZQ) independently extracted data information from the same studies. They then met to review their results and cross-check them. Any discrepancies were resolved through consensus. If consensus could not be reached, a third scholar (QH) provided recommendations. For missing data, we contacted the original authors by email to obtain the necessary information and processed the missing data appropriately; to ensure the accuracy of the data, we did not extract and estimate data from studies in which the key endpoint data were presented only in graphical form. The values could not be obtained from the authors, and were excluded from the quantitative synthesis—ensuring completeness of analysis and reliability of results. After rigorous screening of titles and abstracts, eligible full-text literature was subjected to a detailed review to ensure that it met the inclusion criteria. Data extraction items included: 1) first author and year of publication; 2) sample size, participants (age, gender); 3) intervention, control, and intervention duration, frequency, and periodicity; and 4) outcome measures and differences in pre- and post-intervention scores for each group. The outcome indicators in this study included the Irritable Bowel Syndrome Symptom Severity Scale (IBS-SSS), which covers the dimensions of pain severity, pain duration, abdominal distension, bowel satisfaction, and life interference, with lower scores indicating less severe symptoms; and Irritable Bowel Syndrome Quality of Life Scale (IBS-QOL) was also assessed as a secondary indicator, including the Dysphoria, interference with activity, body image, health worries, food avoidance, social reactions, sexual health, and Relationships, with higher scores indicating a better quality of life (12). The depression scores and anxiety scores were assessed as related indicators of mental health, with lower scores indicating better psychological status.

### 2.5 Assessment of risk of bias

Two researchers (WS and PQ) independently assessed and verified the quality of the literature using the Cochrane 2.0 Manual Randomized Controlled Trial Risk of Bias Assessment Scale (11). The assessment covered seven domains: random sequence generation, allocation concealment, blinding of participants and investigators, blinding of outcome assessors, completeness of outcome reporting, selective reporting, and other biases. Each domain was rated as “high risk”,”low risk” or “unclear”. A risk of bias assessment diagram was generated using R software version 4.3.3.

### 2.6 Methods of standardizing exercise doses

Given the significant differences in intervention modalities and physiological characteristics of different forms of exercise interventions (e.g., running, Pilates, yoga, Baduanjin, and moderate-intensity aerobics), and the large inconsistencies in reporting of exercise frequency and duration in the included studies, the present study did not quantitatively standardize the full range of components of exercise dose. To improve comparability between interventions and to avoid introducing additional bias, with reference to previous methods of network meta-analysis of exercise interventions and non-pharmacological interventions (13), the present study standardized the classification of different exercise modalities mainly based on exercise intensity. Specifically, interventions explicitly described as “moderate-intensity aerobic exercise” in the original study, or interventions with exercise loads equivalent to moderate subjective exertion levels or metabolic equivalents (METs), were standardized to be classified as moderate-intensity aerobic exercise; whereas physical and mental exercises, such as yoga, Pilates, and Baduanjin, were classified as moderate-intensity aerobic exercise because of their unique characteristics in respiratory regulation, neuromodulation, and autonomic activation. autonomic activation, they were included in the network Meta-analysis as independent intervention nodes. Although the frequency and duration of exercise were systematically organized and descriptively summarized during the data extraction process, they were not included as stratification variables or modeling parameters in the statistical analysis because of the lack of relevant data and the large inter-study variations.

### 2.7 Statistical analysis

RevMan 5.4 and R 4.3.3 software were used for Pairwise Meta and network Meta-analysis of the data. The study outcomes comprised continuous variables. Due to variations in the scales and data units used across studies, continuous variables were analyzed using standardized mean difference (SMD) and mean difference (MD), with 95% confidence intervals (CI) calculated. The Q-test was employed for sample analysis.

First, pairwise meta-analysis is used to obtain the heterogeneity (I²) and P-values for direct comparisons between different exercise interventions and the control group. Sensitivity analysis or subgroup analysis may be performed as needed. Subsequently, network meta-analysis is conducted to generate network plots, league tables, and cumulative probability ranking plots. In network meta-analysis, the network evidence diagram visually illustrates direct and indirect comparisons among exercise interventions. Convergence diagnostics were performed for three outcome measures. These diagnostics employed Bayesian fixed-effects models constructed via Markov Chain-Monte Carlo methods, with iterative refinement until satisfactory convergence was achieved. In this study, a potential scale reduction factor (PSRF) between 1 and 1.05 indicated satisfactory model convergence. When the network plot formed closed loops, node splitting was used to test inconsistencies and determine differences between direct and indirect comparison results. A P-value >0.05 indicated consistency between the two sets of results. The ranking of exercise intervention effectiveness will be interpreted based on effect size and SUCRA values, where a higher SUCRA value indicates a greater likelihood that the intervention is the optimal approach. Finally, funnel plots generated using R software will assess potential publication bias among the interventions.

## 3 Results

### 3.1 Literature search results

**Figure 1:**
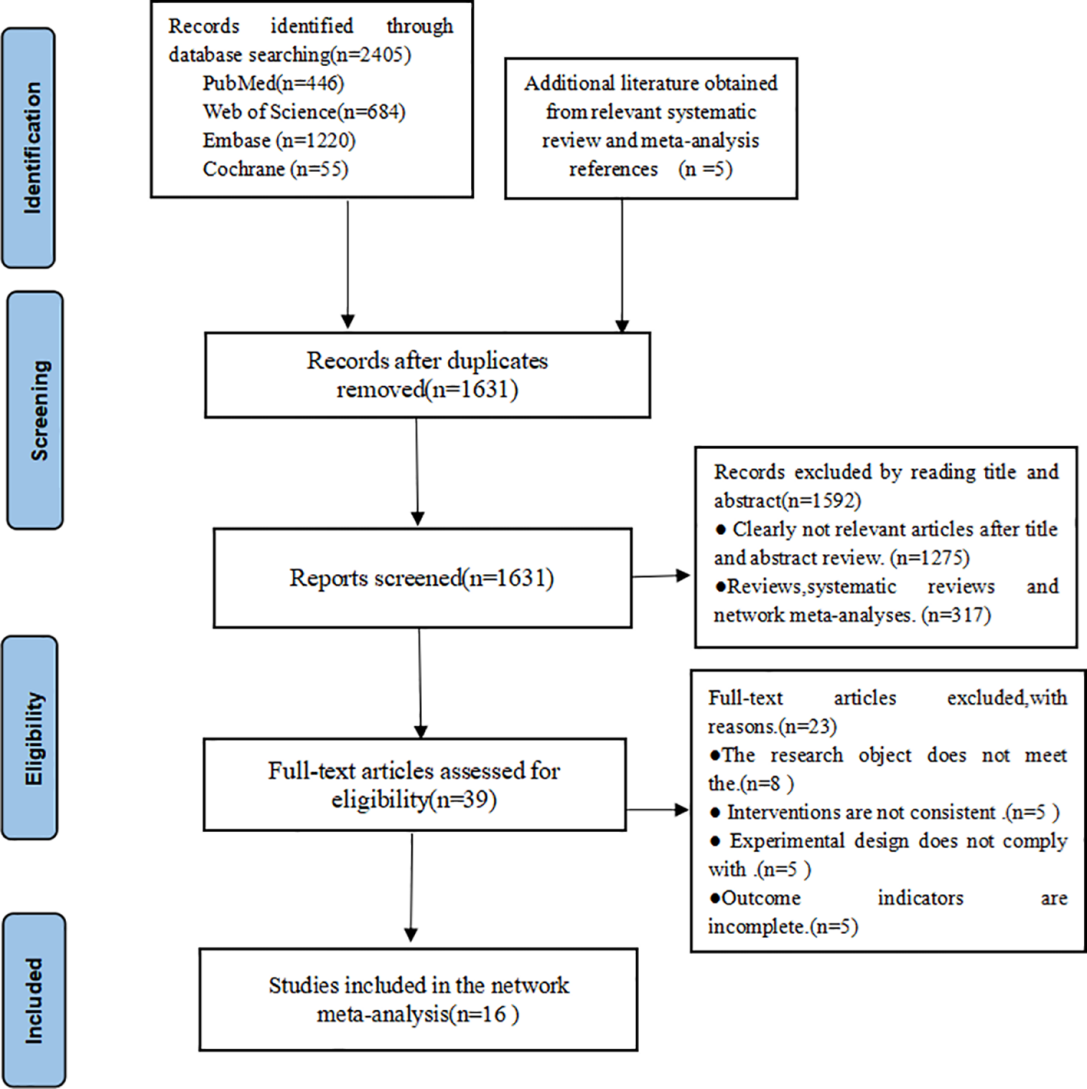
Literature search flow chart.

In this study, a total of 2,405 articles of related literature were retrieved, of which 446 articles in PubMed, 1,220 articles in Embase, 684 articles in Web of Science, 55 articles in Cochrance, and 5 articles obtained from other resources, and 1,631 articles were left after eliminating duplicates, and 1,592 articles were eliminated by reading the titles and abstracts (initial screening), read full-text rescreening of 39 randomized controlled papers; twenty-three papers were excluded based on incompleteness of the study population, intervention, trial design and outcome indicators, and 16 papers were finally included, as shown in **Figure 1**.

### 3.2 Basic characteristics of included studies

A total of 16 independent samples were included from 16 papers, which were published from 2004 to 2025, with a total of 943 subjects in the experimental and control groups, 475 in the experimental group, and 468 in the control group. The main interventions used in the experimental group were five types of yoga exercise, Pilates exercise, moderate intensity aerobic exercise, Baduanjin exercise, and running exercise, and the control group was mainly conventional treatment without any regularity, as shown in **Table 1**.

**Table 1:** Basic characteristics of the included literature.

Author	Country or region	Sex: M/F(E/C)	Age: (M ± SD)/year	IBS Type	Intervention period	Intervention frequency	Intervention		Outcomes
							E	C	
Devi D 2025 (14)	India	E:73/12 C:56/24	E:30.28 ± 7.73 C:32.36 ± 8.19	IBS-C, IBS-D, IBS-M	12 weeks	5time/week	Yoga + conventional medication treatment	Conventional medical treatment	① 、③ 、④ 、1a 、1b 、1c 、1d 、1e 、
Allam D 2024 (15)	Egypt	E:0/30 C:0/30	E:29.4 ± 7.66 C:30,33 ± 8,63	IBS-C	8weeks	2time/week	Pilates+dietary advice	dietary advice	① 、③ 、④ 、② 、1a 、1b 、1c 、1d 、
Chao WC 2024 (16)	Taiwan, China	E:3/7 C:3/8	E:36.5 ± 6.8 C:37.9 ± 4.7	ND	6weeks	3time/week	Yoga+probiotics	probiotics	②
D’Silva A 2022 (17)	Canada	E:2/36 C:4/37	E:43.5 ± 12.9 C:47.1 ± 14.8	ND	8weeks	1time/week	Yoga	Online education courses	②③④
Zhao SR 2019 (18)	China	E:7/21 C:7/22	E:33.75 ± 2.57 C:36.86 ± 2.54	ND	6,12,24 weeks	4time/week	Baduanjin+conventional medical treatment	conventional treatment	①
Tavakoli T 2019 (19)	Iran	E:19 C:18	E:33.1 ± 9.49 C:31.72 ± 9.02	ND	7weeks	ND	Yoga+symptomatic treatment	symptomatic treatment	④
Fani M 2019 (20)	Iran	E:0/10 C:0/10	E:29.1 ± 6.8 C:32.7 ± 10.27	ND	6weeks	3time/week	Running	conventional treatment	①②
Schumann D 2018 (21)	Germany	E:2/28 C:5/24	E:56.33 ± 8.50 C:53.52 ± 10.47	ND	12,24weeks	2time/week	Yoga	Low-FODMAP diet	① 、② 、1a 、1b 、1c 、1d 、1e 、2a-2h
Taneja I 2004 (22)	India	E:9/0 C:13/0	30.9 ± 6.79	IBS-D	4,8weeks	Unclear	Yoga	conventional treatment	④
Yishen Liang 2023 (23)	China	E:20/10 C:18/12	E:40.5 ± 6.1 C:41.7 ± 5.8	IBS-D	8weeks	10time/week, 10day/time	Baduanjin+Acupuncture Point Implantation	Bifidobacterium Triple Live Bacteria Capsules	②
A.J.Daley 2010 (24)	United Kingdom	E:6/22 C:9/19	E:22 ± 78.6 C:19 ± 67.9	ND	12weeks	30minutes/week	Moderate-intensity walking exercise	conventional treatment	② 、1a 、
Yichong Feng 2010 (25)	China	E:17/13 C:19/11	E:66.53 ± 3.32 C:66.47 ± 3.17	IBS-C	12weeks	10time/week	Baduanjin+tegaserodmedical treatment	Tegaserod medication therapy	1c
Elisabet J 2010 (26)	Sweden	E:41/9 C:40/12	E:36 years old(18 to 65 years of age) C:38.5 years old(19-65)	IBS-C, IBS-D, IBS-M	12weeks	3-5time/week	Moderate-intensity physical activity	conventional treatment	2c 、2e 、2f 、2g
MARWA M 2020 (27)	Kasr	E:15 C:15	E:31.6 ± 4.579 C;32.13 ± 5.29	ND	4weeks	3time/week	moderate intensity aerobic exercises	Conventional drug therapy	①②
Yue Jia 2016 (28)	China	E:13/17 C:8/12	41.49 ± 10.72	IBS-D	12weeks	3-5time/week	moderate intensity aerobic exercises	Blank control	① 、② 、③ 、④ 、1a 、1c 、1d 、2a-2h
Kavuri V 2015 (29)	USA	E:2/31 C:6/25	E:44.06 ± 13.42 C:45.08 ± 13.28	IBS-C, IBS-D, IBS-M	12weeks	3time/week	Yoga	conventional treatment	②

E, experimental group; C, control group; M, male; F, female; IBS, irritable bowel syndrome; ND, not described; NR, not reported. IBS-C, constipation-predominant IBS

IBS subtypes: IBS-C,constipation-predominant IBS; IBS-D, diarrhea-predominant IBS; IBS-M, mixed-type IBS.

Outcomes:

① IBS-SSS, Irritable Bowel Syndrome Symptom Severity Scale; ② IBS-QOL, Irritable Bowel Syndrome Quality of Life scale; ③Anxiety; ④Depression; 1a=Pain severity; 1b=Duration of pain; 1c=Abdominal distension; 1d=Bowel satisfaction; 1e=Interference with life; 2a=Dysphoria; 2b=:Interference with activity; 2c=Body image; 2d=Health worries; 2e=Food avoidance; 2f=Social reaction; 2g:Sexual; 2h=Relationships.

### 3.3 Risk of bias assessment

**Figure 2:**
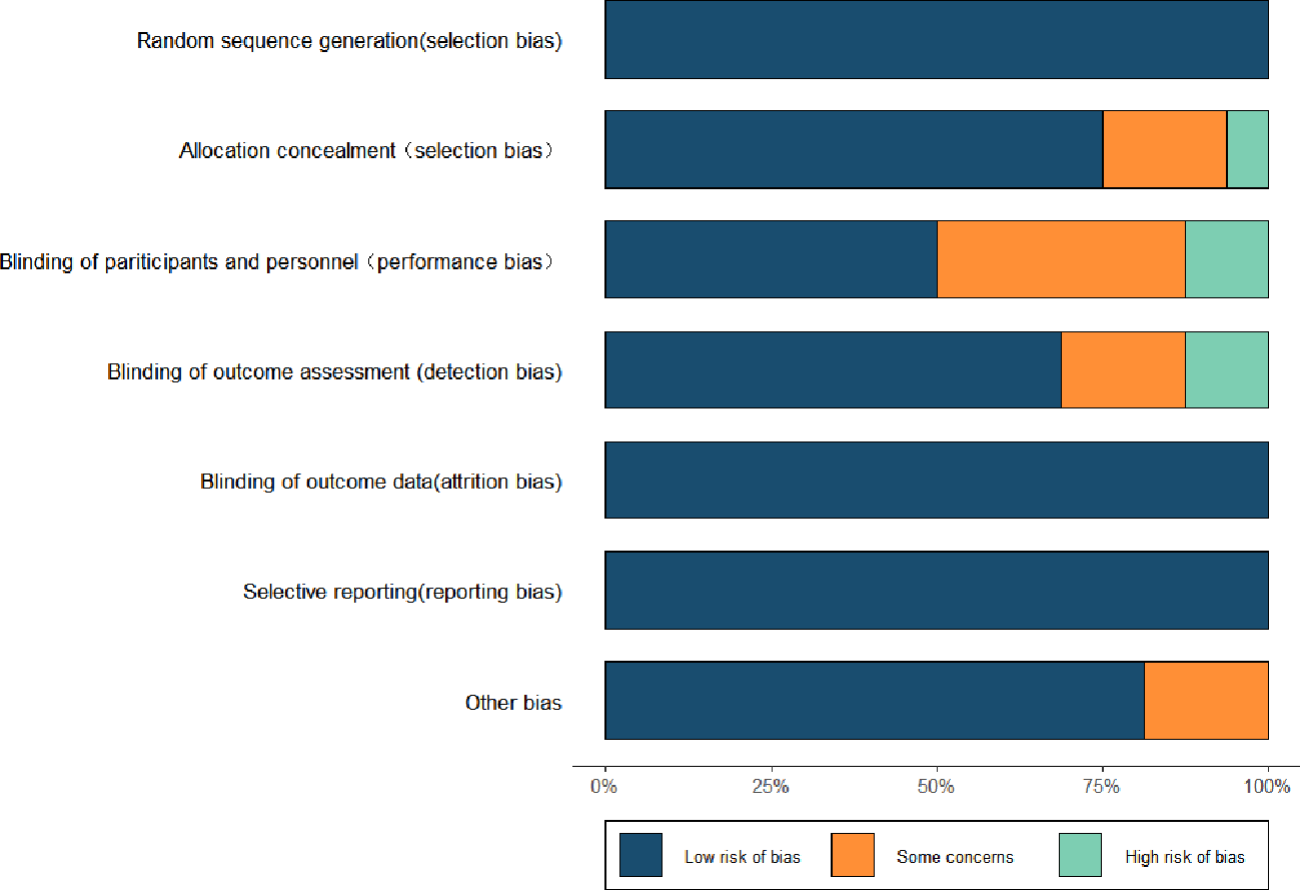
Results of risk of bias evaluation of included studies.

Of the 16 included studies, 3 used computer randomization, 1 used post-it note and dice sampling, 3 used random number tables, 2 used random allocation software for random sampling, and 6 referred only to randomization; all 16 were assessed as low-risk in terms of “randomization method”. Allocation concealment was performed in 16 studies. Eight studies used blinding of implementers and participants, and all eight studies did not mention the evaluation of “blinding of implementers and participants” and rated it as unclear risk. Blinding of outcome assessors in 11 studies and no mention of “blinding of outcome assessors” in the remaining 5 studies were assessed as unclear risk. 16 studies had complete outcome data and were assessed as low risk with respect to “incomplete outcome data”. Sixteen studies had “selective reporting” and were assessed as low risk. 13 studies were assessed as low risk for “other biases” and the remaining 3 studies did not mention “other biases” and were assessed as unclear risk. The remaining 3 studies did not mention “other biases” and were assessed as unclear risk (see **Figure 2**).

### 3.4 Rating directions and effect size definitions

To avoid directional confusion among different outcome measures in the results narrative, this paper provides a unified explanation of the scoring directions and effect size interpretations for each scale. For the IBS-SSS scale and psychological symptom scales (anxiety and depression scores), lower scale scores indicate milder symptoms and better intervention outcomes. Therefore, a negative effect size (SMD or MD < 0) suggests that the exercise intervention may have a beneficial effect compared to the control group. For quality of life-related scales (IBS-QOL), higher scores indicate better quality of life. Thus, a positive effect size (MD > 0) suggests that exercise intervention may improve quality of life. Throughout this paper—in Pairwise meta-analyses, network meta-analyses, and pairwise comparison discussions—the efficacy of different exercise interventions is consistently interpreted based on the aforementioned scoring directions. This ensures consistency in the direction and clinical significance of all outcome results.

Given the observation of relatively large effect sizes in certain outcome measures (e.g., higher absolute values of SMD or MD), this study conducted a retrospective verification of the relevant research. This included: verifying the scoring direction and range of each scale; confirming that outcome measures represented pre-to-post intervention changes or post-intervention endpoint values; rechecking the accuracy of standard deviation and sample size entries; and excluding effect size inflation caused by inconsistent units or scale misuse. The verification results indicated that all relevant effect sizes originated from the data reported in the original literature, with no errors identified in data extraction or scale standardization. Furthermore, sensitivity analyses demonstrated that the direction of pooled effect sizes remained consistent across different effect models, suggesting no significant bias in model selection.

### 3.5 Pairwise meta-analysis

Categorical analyses by indicator, due to the small number of literature included in the study for indicators of depression scores, IBS-SSS scale sub-dimensions (pain severity, pain duration, abdominal distension, bowel satisfaction, and interference with life), and other dimensions related to the IBS-QOL scale (e.g., body image, health worries, food avoidance, relationships) were included in the study. Furthermore, only experimental and control groups were compared, failing to meet the criteria for a network meta-analysis. Therefore, only a pairwise meta-analysis was conducted.

#### 3.5.1 Depression score

Four studies included continuous changes in depression scores among IBS patients following intervention (see **Table 2**). Pairwise meta-analysis revealed substantial heterogeneity (I² = 95%). The pooled effect size indicated [SMD = −0.56, 95% CI: (−0.78, −0.34), Z = 4.90, P < 0.00001]. Research indicates that compared to conventional treatment, exercise demonstrates a statistically significant difference in improving depression scores among IBS patients, exerting a notable impact. Analysis by intervention type revealed that the yoga intervention showed low heterogeneity (I² = 0%, P = 0.34). No significant difference existed between the experimental group and the conventional treatment group post-intervention, with results lacking statistical significance [SMD=-0.20, 95% CI: (-0.45, 0.05), P = 0.12]; Pilates exercise intervention significantly reduced depression scores in IBS patients, with statistically significant results [SMD = -1.26, 95% CI: (-1.82, -0.70), P < 0.00001]; Moderate-intensity aerobic exercise intervention effectively reduced depression scores in IBS patients, with statistically significant differences [SMD = -4.26, 95% CI: (-5.30, -3.22), P < 0.00001]. Significant differences existed in depression scores among IBS patients across different exercise interventions (P < 0.00001), indicating that exercise type is a source of heterogeneity in depression scores among IBS patients.

**Table 2:** Meta-analysis results for depression scores.

Outcome indicator	Heterogeneity test		Meta-analysis		Subgroup differences (P)
	I^2^%	P-value	SMD and 95% CI	P-value	
Depression Score	95	<0.00001	-0.56[-0.78,-0.34]	<0.00001	<0.00001
Yoga	0	0.34	-0.20[-0.45,0.05]	0.12
Pilates	×	×	-1.26[-1.82,-0.70]	<0.00001
MAE	×	×	-4.26[-5.30,-3.22]	<0.00001

MAE, derate-intensity aerobic exercise; ×, Not specified in the literature; P-value, Statistical significance level; SMD, Standardized Mean Difference, represents the effect size between experimental and control groups. CI, Confidence Interval, indicating the range within which the true effect size is likely to fall with 95% certainty. A negative SMD, indicates a better outcome in the intervention group compared to the control group. A P-value less than 0.05 indicates that the result is statistically significant.

#### 3.5.2 IBS-SSS scale sub-dimensions

This meta-analysis included 23 studies and examined the effects of interventions on each subscale of the IBS-SSS scale in patients with IBS (see **Table 3**). Results showed no significant overall difference in Pain severity (I²=91%), with a mean difference (MD) of 0.18 (95% CI: -1.50 to 1.86; P > 0.05) and no statistically significant differences in the results; analysis by intervention type showed that Pilates [MD = -17.90, 95% CI: (-24.74, -11.06), P < 0.00001] and moderate-intensity aerobic exercise [MD = 2.26, 95% CI: (0.32, 4.20), P = 0.02] significantly reduced pain severity in IBS patients, whereas yoga showed no significant effect [MD=-2.26, 95% CI: (-6.08, 1.56), P = 0.25]; significant differences existed between exercise modalities (P<0.00001). Duration of pain (I²=73%): Exercise interventions showed overall significant improvement [MD=-5.16, 95% CI: (-9.16, -1.17), P<0.05]; Pilates demonstrated the most pronounced effect [MD=-13.33, 95% CI: (-20.72, -5.94), P = 0.0004], while yoga showed no significant difference [MD=-1.79, 95% CI: (-6.54, 2.96), P = 0.25]; differences between exercise types were significant (P = 0.01). Abdominal distension (I²=81%): The pooled effect showed significant differences [MD = 0.87, 95% CI: (0.52, 1.22), P<0.05]. Analyses of different exercise interventions indicated that Pilates [MD=-11.80, 95% CI: (-21.29, -2.31), P = 0.01], Baguazhang [MD = 0.93, 95% CI: (0.58, 1.28), P<0.00001], and moderate-intensity aerobic exercise [MD=-14.83, 95% CI: (-24.10, -5.56), P = 0.002] effectively improved abdominal distension scores in IBS patients, whereas yoga showed no significant effect [MD=-1.56, 95% CI: (-5.15, 2.03), P = 0.39]; with significant differences between exercise types (P = 0.0002). Bowel satisfaction (I²=54%): Overall effects showed exercise interventions improved [MD=-4.78, 95% CI: (-7.72, -1.84), P<0.05]; with yoga [MD = -4.21, 95% CI: (-7.51, -0.90), P = 0.01] and Pilates [MD = -11.57, 95% CI: (-19.58, -3.56), P = 0.005] showed significant improvements, while aerobic exercise had no clear effect [MD = 1.42, 95% CI: (-9.34, 12.18), P = 0.80]; no statistically significant differences existed between exercise types (P = 0.13). Interference with life (I²=0%): No significant overall difference was observed [MD=-4.93, 95% CI: (-8.50, -1.36), P>0.05]. Analysis of different exercise interventions showed yoga significantly improved this score [MD = -4.93, 95% CI: (-8.50, -1.36), P = 0.007], while other exercise modalities had no significant effect. Overall, exercise interventions improved selected subscales of the IBS-SSS in IBS patients. Pilates demonstrated particularly strong efficacy in alleviating pain severity (MD = -17.90), pain duration (MD = -13.33), and abdominal distension (MD = -11.80). Moderate-intensity aerobic exercise significantly alleviated pain (MD = 2.26) and abdominal distension (MD=-14.83); yoga demonstrated limited effects only in certain dimensions (e.g., Bowel satisfaction, Interference with life). Differences in exercise modalities likely represent the primary source of heterogeneity in IBS-SSS subscale outcomes.

**Table 3:** Pairwise meta-analysis results for IBS-SSS scale sub-dimensions.

Outcome indicator	Heterogeneity test		Meta-analysis		Subgroup differences (P)
	I^2^%	P-value	MD and 95% CI	P-value	
Pain severity	91	<0.00001	0.18[-1.50,1.86]	0.83	<0.00001
Yoga	85	0.001	-2.26[-6.08,1.56]	0.25
Pilates	×	×	-17.90[-24.74,-11.06]	<0.00001
MAE	92	0.0006	2.26[0.32,4.20]	0.02
Duration of pain	73	0.01	-5.16[-9.16,-1.17]	0.01	0.01
Yoga	57	0.10	-1.79[-6.54,2.96]	0.46
Pilates	×	×	-13.33[-20.72,-5.94]	0.0004
Abdominal distension	81	<0.0001	0.87[0.52,1.22]	<0.00001	0.0002
Yoga	69	0.04	-1.56[-5.15,2.03]	0.39
Pilates	×	×	-11.80[-21.29,-2.31]	0.01
Baduanjin	×	×	0.93[0.58,1.28]	<0.00001
MAE	×	×	-14.83[-24.10,-5.56]	0.002
Bowel satisfaction	54	0.07	-4.78[-7.72,-1.84]	0.001	0.13
Yoga	57	0.10	-4.21[-7.51,-0.90]	0.01
Pilates	×	×	-11.57[-19.58,-3.56]	0.005
MAE	×	×	1.42[-9.34,12.18]	0.80
Interference with life	0	0.63	-4.93[-8.50,-1.36]	0.007	×
Yoga	0	0.63	-4.93[-8.50,-1.36]	0.007

MAE, Moderate-intensity aerobic exercise; ×, Not specified in the literature; CI, Confidence Interval; MD, mean difference; P-value, Statistical significance level.

#### 3.5.3 IBS-QOL scale sub-dimensions

A meta-analysis of the 27 studies included in this research examined each subscale of the IBS-QOL scale (see **Table 4**). Results indicated: For the Dysphoria subscale (I²=76%), the pooled effect size demonstrated that exercise intervention outperformed conventional treatment [MD = 2.33, 95% CI: (-0.58, 5.25), P<0.05], revealing a statistically significant difference. Analysis by exercise type revealed no significant difference for yoga intervention, whereas moderate-intensity aerobic exercise significantly improved Dysphoria scores [MD = 5.22, 95% CI: (1.67, 8.77), P = 0.004]; Comparisons of Dysphoria scores across IBS-QOL subdimensions revealed differences between exercise interventions, suggesting exercise type may be a source of heterogeneity (P = 0.005). Interference with activity (I²=91%), The results showed that exercise interventions significantly improved this score [MD = 7.19, 95% CI:(3.77,10.60), P<0.05], with a clear statistical difference; among them, aerobic exercise had a more pronounced effect [MD = 15.42, 95% CI:(10.51,20.33), P<0.00001]; with significant differences between exercise modalities (P < 0.00001). The comprehensive effect results for Body image (I²=56%) and Health worries (I²=88%) indicated that exercise significantly influenced IBS-QOL subscale scores for Body image and Health worries in IBS patients, with statistically significant differences. Categorization by exercise type showed that moderate-intensity aerobic exercise also produced significant improvements compared to conventional treatment, whereas yoga intervention yielded no significant effects. Overall differences for Food avoidance (I²=11%) were not significant. Analysis by exercise type showed both yoga and moderate-intensity aerobic exercise produced statistically significant results, with no significant between-group differences. Overall effects for Social reaction (I²=0%) and Sexual (I²=54%) were not significant. Analysis by intervention type indicated moderate-intensity aerobic exercise demonstrated some improvement in Social reaction. Relationships (I²=67%) showed a significant overall effect [MD = 3.63, 95% CI:(0.77,6.50), P<0.05]; subgroup analysis by exercise type indicated moderate-intensity aerobic exercise produced stronger effects (P = 0.001). Overall, exercise interventions improved multiple subscales of the IBS-QOL in IBS patients. Moderate-intensity aerobic exercise notably alleviated dysphoria, activity interference, health concerns, body image, and interpersonal relationships, while yoga showed limited effects on specific subscales. Exercise type likely contributes significantly to the heterogeneity observed across IBS-QOL subscales.

**Table 4:** Pairwise meta-analysis results for IBS-QOL scale sub-dimensions.

Outcome indicator	Heterogeneity test		Effect model	Meta-analysis		Subgroup differences (P)
	I^2^%	P-value		MD and 95% CI	P-value	
Dysphoria	76	0.02	FE	2.33[-0.58,5.25]	0.12	0.005
Yoga	0	0.49	FE	-3.66[-8.78,1.45]	0.16
MAE	×	×	FE	5.22[1.67,8.77]	0.004
Interference with activity	91	<0.0001	FE	7.19[3.77,10.60]	<0.0001	<0.00001
Yoga	6	0.30	FE	-0.53[-5.29,4.22]	0.83
MAE	×	×	FE	15.42[10.51,20.33]	<0.00001
Body image	56	0.08	FE	6.35[3.20,9.51]	<0.0001	0.01
Yoga	0	0.39	FE	0.61[-4.99,6.21]	0.83
MAE	0	0.68	FE	9.03[5.21,12.86]	<0.00001
Health worries	88	0.0002	FE	5.90[3.26,8.54]	<0.0001	<0.0001
Yoga	35	0.22	FE	-3.96[-9.55,1.63]	0.17
MAE	×	×	FE	8.72[5.73,11.71]	<0.00001
Food avoidance	11	0.34	FE	9.06[5.75,12.37]	<0.00001	0.22
Yoga	29	0.23	FE	13.68[5.52,21.83]	0.001
MAE	0	0.49	FE	8.15[4.52,11.77]	<0.0001
Social reaction	0	0.51	FE	2.63[-0.26,5.52]	0.07	0.16
Yoga	0	0.60	FE	-0.44[-5.59,4.71]	0.87
MAE	0	0.78	FE	4.03[0.54,7.52]	0.02
Sexual	54	0.09	FE	1.45[-2.33,5.24]	0.45	0.01
Yoga	0	0.64	FE	-7.07[-14.75,0.61]	0.07
MAE	0	0.87	FE	4.19[-0.16,8.54]	0.06
Relationships	67	0.05	FE	3.63[0.77,6.50]	0.01	0.02
Yoga	0	0.75	FE	-5.04[-12.61,2.53]	0.19
MAE	×	×	FE	5.08[1.99,8.17]	0.001

FE, Fixed effects mode; MAE, Moderate-intensity aerobic exercise; ×,Not specified in the literature; CI, Confidence Interval; MD, Mean Difference; P-value, Statistical significance level.

### 3.6 Sensitivity analysis

Sensitivity analyses were conducted by eliminating sources of heterogeneity. Due to varying heterogeneity among the included outcome measures, different effect models were applied to analyze the data, thereby assessing the stability of the study findings. By comparing effect size values obtained through different effect models, the combined effect sizes for each risk factor remained consistent without significant variation. This indicates that the meta-analysis results are relatively stable; conversely, instability would necessitate identifying the factors contributing to such inconsistency. Sensitivity testing results (see **Table 5**) showed that comparing effect sizes from fixed-effect and random-effect models yielded similar combined effect sizes for each risk factor. The fixed-effect model fell within the confidence interval of the random-effect model, with no differential changes in outcomes. This indicates stable meta-analysis results with low sensitivity and good stability.

**Table 5:** Pairwise meta sensitivity analysis.

Outcome	Effect model	Effect size	95% Confidence interval	Effect model	Effect size	95% Confidence interval
Depression Score	FE	SMD=-0.56	[-0.78,-0.34]	RE	SMD=-1.34	[-2.50, -0.19]
Yoga	FE	SMD=-0.20	[-0.45,0.05]	RE	SMD=-0.20	[-0.45, 0.05]
Pilates	FE	SMD=-1.26	[-1.82,-0.70]	RE	SMD=-1.26	[-1.82, -0.70]
MAE	FE	SMD=-4.26	[-5.30,-3.22]	RE	SMD=-4.26	[-5.30,-3.22]
Pain severity	FE	MD=0.18	[-1.50, 1.86]	RE	MD=-3.66	[-11.76, 4.44]
Yoga	FE	MD=-2.26	[-6.08, 1.56]	RE	MD=2.78	[-10.07, 15.62]
Pilates	FE	MD=-17.90	[-24.74,-11.06]	RE	MD=-17.90	[-24.74,-11.06]
MAE	FE	MD=2.26	[0.32,4.20]	RE	MD=-5.77	[-24.16,12.61]
Duration of pain	FE	MD=-5.16	[-9.16,-1.17]	RE	MD=-2.02	[-11.50,7.45]
Yoga	FE	MD=-1.79	[-6.54, 2.96]	RE	MD=2.80	[-8.09,13.69]
Pilates	FE	MD=-13.33	[-20.72,-5.94]	RE	MD=-13.3	[-20.72,-5.94]
Abdominal distension	FE	MD=0.87	[0.52,1.22]	RE	MD=-2.61	[-7.85,2.63]
Yoga	FE	MD=-1.56	[-5.15, 2.03]	RE	MD=2.53	[-6.53, 11.58]
Pilates	FE	MD=-11.80	[-21.29,-2.31]	RE	MD=-11.80	[-21.29,-2.31]
Baduanjin	FE	MD=0.93	[0.58, 1.28]	RE	MD=0.93	[0.58, 1.28]
MAE	FE	MD=-14.83	[-24.10,-5.56]	RE	MD=-14.83	[-24.10,-5.56]
Bowel satisfaction	FE	MD=-4.78	[-7.72,-1.84]	RE	MD=-3.06	[-8.66, 2.54]
Yoga	FE	MD=-4.21	[-7.51,-0.90]	RE	MD=-0.88	[-8.68,6.92]
Pilates	FE	MD=-11.57	[-19.58,-3.56]	RE	MD=-11.57	[-19.58,-3.56]
MAE	FE	MD=1.42	[-9.34,12.18]	RE	MD=1.42	[-9.34,12.18]
Interference with life	FE	MD=-4.93	[-8.50,-1.36]	RE	MD=-4.93	[-8.50,-1.36]
Yoga	FE	MD=-4.93	[-8.50,-1.36]	RE	MD=-4.93	[-8.50,-1.36]
Dysphoria	FE	MD=2.33	[-0.58,5.25]	RE	MD=-0.25	[-7.25,6.74]
Yoga	FE	MD=-3.66	[-8.78,1.45]	RE	MD=-3.66	[-8.78,1.45]
MAE	FE	MD=5.22	[1.67,8.77]	RE	MD=5.22	[1.67,8.77]
Interference with activity	FE	MD=7.19	[3.77,10.60]	RE	MD=4.90	[-6.75,16.55]
Yoga	FE	MD=-0.53	[-5.29,4.22]	RE	MD=-0.54	[-5.46,4.37]
MAE	FE	MD=15.42	[10.51,20.33]	RE	MD=15.42	[10.51,20.33]
Body image	FE	MD=6.35	[3.20,9.51]	RE	MD=4.63	[-1.51,10.78]
Yoga	FE	MD=0.61	[-4.99,6.21]	RE	MD=0.61	[-4.99,6.21]
MAE	FE	MD=9.03	[5.21,12.86]	RE	MD=9.03	[5.21,12.86]
Health worries	FE	MD=5.90	[3.26,8.54]	RE	MD=0.54	[-9.84,10.91]
Yoga	FE	MD=-3.96	[-9.55,1.63]	RE	MD=-4.12	[-11.07,2.84]
MAE	FE	MD=8.72	[5.73,11.71]	RE	MD=8.72	[5.73,11.71]
Food avoidance	FE	MD=9.06	[5.75,12.37]	RE	MD=9.40	[5.15,13.66]
Yoga	FE	MD=13.68	[5.52,21.83]	RE	MD=13.87	[4.14,23.60]
MAE	FE	MD=8.15	[4.52,11.77]	RE	MD=8.15	[4.52,11.77]
Social reaction	FE	MD=2.63	[-0.26,5.52]	RE	MD=2.63	[-0.26,5.52]
Yoga	FE	MD=-0.44	[-5.59,4.71]	RE	MD=-0.44	[-5.59,4.71]
MAE	FE	MD=4.03	[0.54,7.52]	RE	MD=4.03	[0.54,7.52]
Sexual	FE	MD=1.45	[-2.33,5.24]	RE	MD=-0.68	[-7.74,6.39]
Yoga	FE	MD=-7.07	[-14.75,0.61]	RE	MD=-7.07	[-14.75,0.61]
MAE	FE	MD=4.19	[-0.16,8.54]	RE	MD=4.19	[-0.16,8.54]
Relationships	FE	MD=3.63	[0.77,6.50]	RE	MD=-0.30	[-8.33,7.72]
Yoga	FE	MD=-5.04	[-12.61,2.53]	RE	MD=-5.04	[-12.61,2.53]
MAE	FE	MD=5.08	[1.99,8.17]	RE	MD=5.08	[1.99,8.17]

RE, random effects model; FE, Fixed-effects model; MAE, Moderate-intensity aerobic exercise; SMD, Standardized Mean Difference; MD, Mean Difference; 95% CI , 95% Confidence Interval. SMD is a measure of the effect size, and the MD indicates the mean difference between groups. The 95% CI shows the range within which the true effect size is likely to fall with 95% confidence. Effect Model: Indicate whether the Fixed Effect Model (FE) or Random Effect Model (RE) is used. Effect Size (SMD and MD): SMD is used when comparing standardized differences between groups, while MD is used for raw mean differences. 95% Confidence Interval (CI): The range within which the true effect size is expected to lie with 95% confidence.

### 3.7 Network meta-analysis

#### 3.7.1 Network relationship diagram

**Figure 3:**
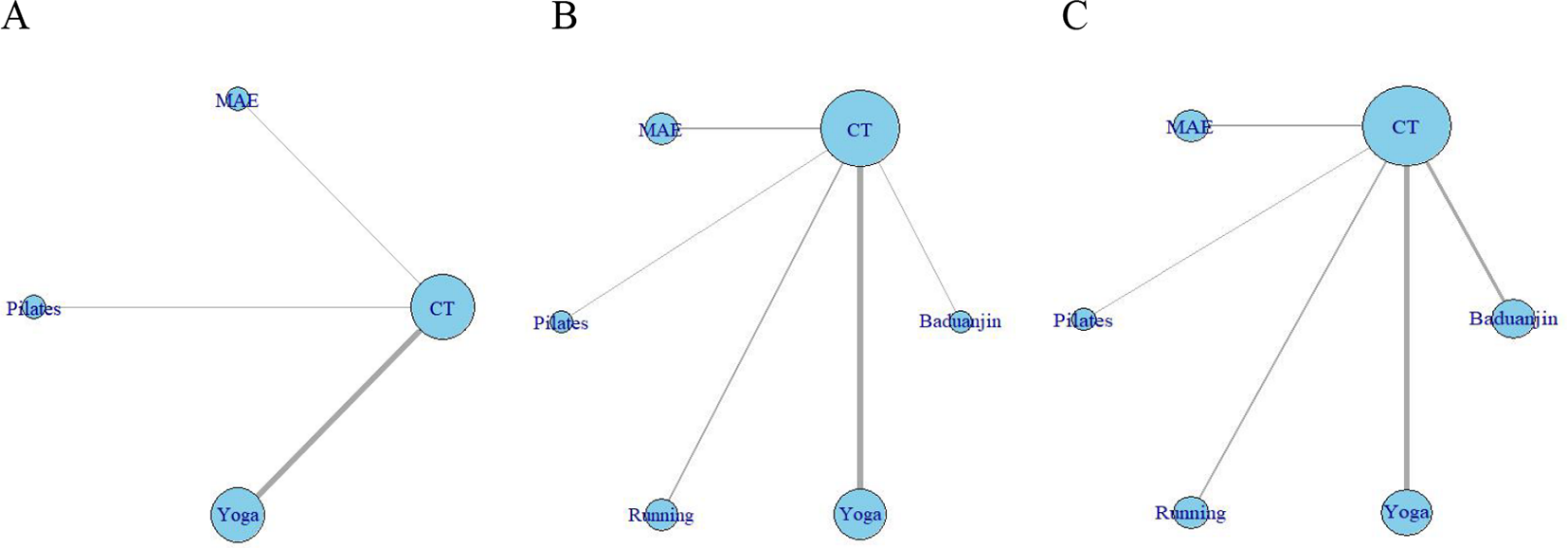
Evidence network diagram for various exercise interventions. **(A)** Anxiety score; **(B)** IBS-QOL scale; **(C)** IBS-SSS scale; MAE, Moderate-intensity aerobic exercise;CT, Conventional treatment.

Using R software to plot a network relationship diagram (see **Figure 3**), seven studies reported anxiety score indicators, involving four exercise interventions and conventional treatment measures, forming seven two-arm studies. Eleven studies reported IBS-QOL scale indicators, involving six exercise interventions and conventional treatment measures, forming eleven two-arm studies. Twelve studies reported IBS-SSS scale indicators, involving six exercise interventions and conventional treatment measures, forming twelve two-arm studies.

#### 3.7.2 Anxiety score

**Figure 4:**
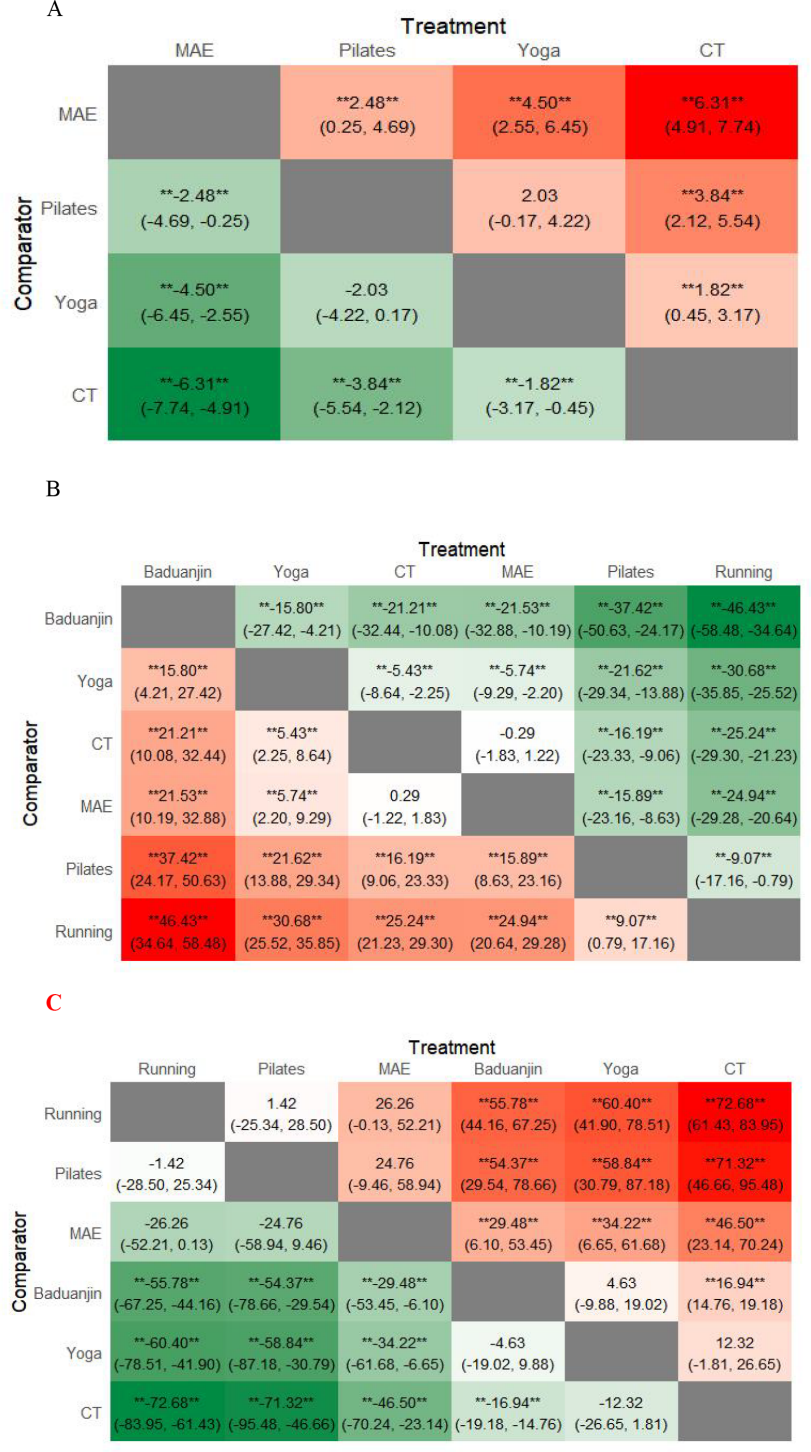
Network meta-analysis results. **(A)** Anxiety score; **(B)** IBS-QOL scale; **(C)** IBS-SSS scale;CT, Conventional treatment; MAE, Moderate-intensity aerobic exercise.(**)=represent statistical significance; **=indicates p < 0.05;Each value in the cells represents the effect size between two treatments; larger values indicate a more significant effect.

A network meta-analysis of the included studies yielded six pairwise comparisons. As shown in **Figure 3**, eight studies reported anxiety score levels in IBS patients, with a total sample size of 443. The network meta-analysis results indicated that yoga [SMD = -1.82, 95% CI: (-3.17, -0.45), P < 0.05], Pilates [SMD = -3.84, 95% CI: (-2.12, -5.54), P < 0.05], and moderate-intensity aerobic exercise [SMD = -6.31, 95% CI: (-4.91, -7.74), P < 0.05] demonstrated statistically significant differences compared to the conventional treatment group. Pairwise comparisons revealed that yoga [SMD = 4.50, 95% CI: (2.55, 6.45), P<0.05], Pilates [SMD = 2.48, 95% CI: (0.25, 4.69), P<0.05] both demonstrated superiority over moderate-intensity aerobic exercise in significantly improving anxiety score levels among IBS patients. Differences between the remaining pairwise comparisons were not statistically significant (P>0.05) (See **Figure 4**).

**Figure 5:**
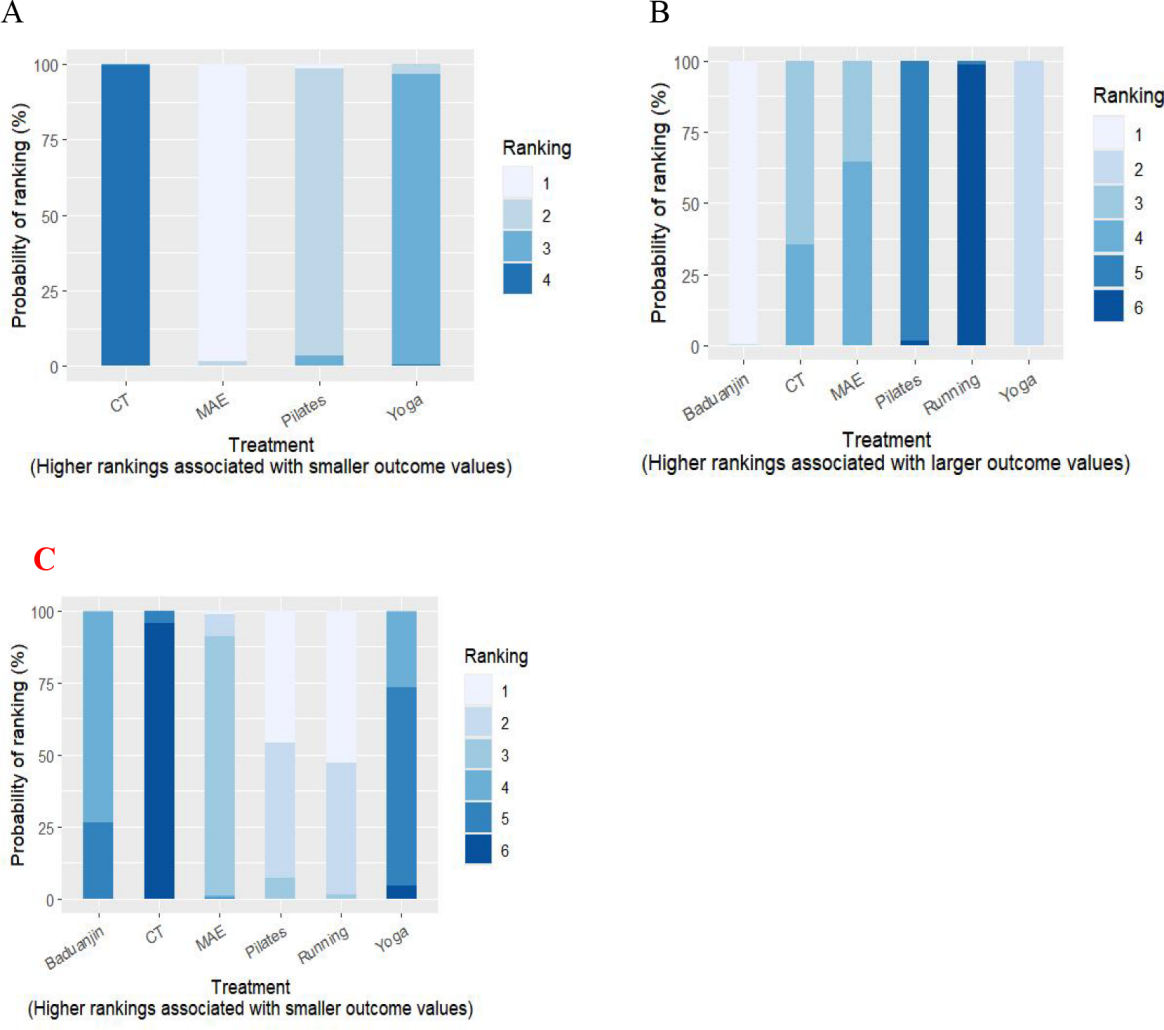
Area under the cumulative probability ranking curve. **(A)** Anxiety score; **(B)** IBS-QOL scale; **(C)** IBS-SSS scale; CT, Conventional treatment; MAE, Moderate-intensity aerobic exercise. Ranking: The rank of each treatment based on its outcome, with 1 being the highest rank and 6 being the lowest rank.Probability of Ranking: The likelihood of each treatment receiving a specific rank.

According to the SUCRA probability ranking, moderate-intensity aerobic exercise (0.4%) > Pilates (33.9%) > yoga (65.8%) > conventional treatment (99.9%). (See **Figure 5**).

#### 3.7.3 IBS-QOL scale

A network meta-analysis of the included studies yielded 15 pairwise comparisons. As shown in **Figure 3**, 11 studies reported on the IBS-QOL scale for IBS patients, with a total sample size of 545. The results of the network meta-analysis showed that running [MD = -25.24, 95% CI: (-29.30, -21.23), P < 0.05], Pilates [MD = -16.19, 95% CI: (-23.33, -9.06), P < 0.05], and Baduanjin exercises [MD = 21.21, 95% CI: (10.08, 32.44), P < 0.05], and yoga [MD = 5.43, 95% CI: (2.25, 8.64), P < 0.05]. Compared with the conventional treatment group, all four exercise intervention methods significantly improved the IBS-QOL Scale (P<0.05). Furthermore, moderate-intensity aerobic exercise [MD = −0.29, 95% CI: (−1.83, 1.22), P > 0.05] showed no statistically significant difference compared with conventional treatment. Pairwise comparisons revealed that Pilates exercise was superior to running [MD = 9.07, 95% CI: (0.79, 17.16), P<0.05]; moderate-intensity aerobic exercise was superior to running [MD = 24.94, 95% CI: (20.64, 29.28), P<0.05] and Pilates [MD = 15.89, 95% CI: (8.63, 23.16), P<0.05]; Yoga outperformed running [MD = 30.68, 95% CI:(25.52, 35.85), P<0.05] and Pilates [MD = 21.62, 95% CI:(13.88, 29.34), P < 0.05], and moderate-intensity aerobic exercise [MD = 5.74, 95% CI: (2.20, 9.29), P < 0.05]; Baduajin was superior to running [MD = 46.43, 95% CI: (34.64, 58.48), P < 0.05], Pilates [MD = 37.42, 95% CI: (24.17, 50.63), P<0.05], moderate-intensity aerobic exercise [MD = 21.53, 95% CI: (10.19, 32.88), P<0.05], yoga [MD = 15.80, 95% CI: (4.21, 27.42), P<0.05]. No statistically significant differences were observed between the remaining exercise groups when compared pairwise (P>0.05) (See **Figure 4**).

According to the SUCRA ranking results: Baduanjin (99.9%) > Yoga (80.1%) > Conventional treatment (52.8%) > Moderate-intensity aerobic exercise (47.3%) > Pilates (19.6%) > Running (0.4%). (See **Figure 5**).

#### 3.7.4 IBS-SSS scale

A network meta-analysis of the included studies produced 15 pairwise comparisons (see **Figure 3**). A total of 10 studies reported on the IBS-SSS scale, with a sample size of 634. The results of the network meta-analysis showed that compared to the conventional treatment group, running [MD = -72.68, 95% CI:(-83.95, -61.43), Pilates [MD = -71.32, 95% CI:(-95.48, -46.66), P<0.05], moderate-intensity aerobic exercise [MD = -46.50, 95% CI:(-70.24, -23.14), P < 0.05], and Baduanjin [MD = -16.94, 95% CI:(-19.18, -14.76), P < 0.05] were the four types of exercise interventions that showed a significant effect (P < 0.05). A pairwise comparison showed that Baduanjin, yoga, and moderate-intensity aerobic exercise were significantly better than running, Pilates, and moderate-intensity aerobic exercise in improving the IBS-SSS scale (P < 0.05) (See **Figure 4**).

According to the results of SUCRA probability ranking, running (90.3%) > Pilates (87.6%) > moderate intensity aerobic (61.8%) > Baduanjin (35%) > Yoga (24.3%) > conventional therapy (1%). (See **Figure 5**).

### 3.8 Publication bias

**Figure 6:**
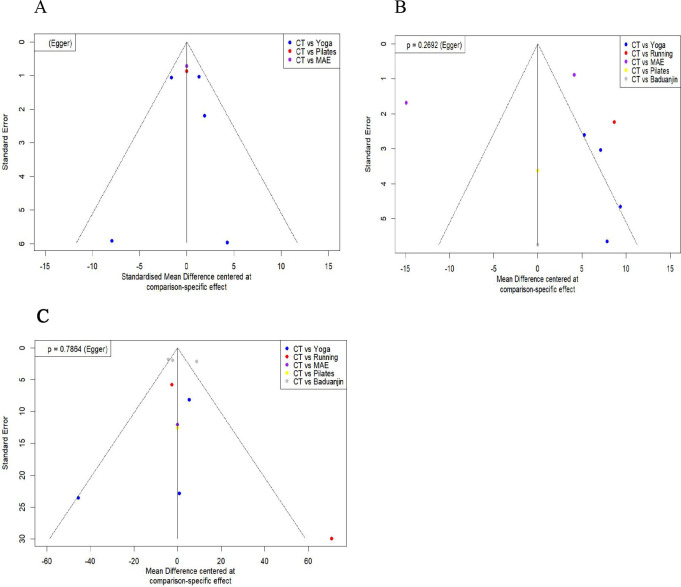
Comparison-correction funnel diagram. **(A)** Anxiety score; **(B)** IBS-QOL scale; **(C)** IBS-SSS scale; CT: Conventional treatment (colored yellow in all plots); MAE, Moderate-intensity aerobic exercise(colored red in all plots).Yoga: (colored blue in all plots); Pilates: (colored purple in all plots); Running: (colored green in all plots);Baduanjin: (colored light orange in panel **(C)** p-value (Egger): Indicates the p-value from the Egger test for funnel plot asymmetry. The Standardized Mean Difference (SMD) or Mean Difference (MD) is shown along the x-axis, while the Standard Error is shown along the y-axis.

In the included studies, funnel plots were constructed for anxiety score levels, IBS-QOL scale, and IBS-SSS scale among IBS patients to assess publication bias. Results showed that the plots were largely symmetrical around zero across all studies (P>0.05), indicating no evidence of publication bias (see **Figure 6**).

### 3.9 Convergence diagnostics and inconsistency tests

Convergence diagnostics were performed on three outcome measures: anxiety scores, the IBS-QOL scale, and the IBS-SSS scale. Results showed that the PSRF values for each outcome measure were close to 1 (range 1.00–1.01), indicating that the model had essentially converged and that the analysis results were relatively reliable. Additionally, node splitting analysis was applied to direct and indirect comparisons within the network relationships to conduct inconsistency tests. Results showed no statistically significant differences (P>0.05) between direct and indirect comparisons for anxiety scores, IBS-QOL scale, and IBS-SSS scale. This indicates good consistency between direct and indirect comparison results.

## 4 Discussion

In terms of psychological symptoms, we found selective effects of different exercise interventions. Pilates and moderate-intensity aerobic exercise showed significant improvement in depressive symptoms, whereas yoga was not as effective. However, for anxiety symptoms, yoga, Pilates and moderate-intensity aerobics showed significant benefits, with moderate-intensity aerobics having the best effect. This difference may be related to the fact that Pilates and moderate-intensity aerobic exercise alleviate anxiety and depressive symptoms by activating the parasympathetic nervous system and modulating the hypothalamic-pituitary-adrenal (HPA) axis (30–32), Aerobic exercise has a clear physiological basis in relieving generalized anxiety, such as promoting blood circulation, regulating autonomic tone (15), and ultimately exerting a moderating effect by reducing inflammatory responses, improving intestinal barrier function and regulating intestinal microecology (18).

In terms of quality of life, exercise interventions can have a positive impact across multiple sub-dimensions of IBS-QOL, but the magnitude of improvement is not entirely consistent across exercise modalities. Previous studies have pointed out that impaired quality of life is closely related to psychological burden, and therefore improvement may be achieved more through relieving psychological stress (32, 33). Monda et al. (32)pointed out that impaired quality of life dimensions such as activity limitation, body image, and health worries in patients with IBS are closely related to the degree of anxiety and depression, and therefore improvement in quality of life with exercise intervention may be achieved more by indirectly by relieving psychological burdens rather than relying solely on direct interventions for gastrointestinal symptoms.

In terms of symptom severity, different forms of exercise showed an uneven pattern of improved performance across the IBS-SSS sub-dimensions. Some exercises may alleviate symptoms by improving gut motility and autonomic regulation (15, 34). Previous studies have shown that moderate-intensity aerobic exercise may be involved in symptomatic relief by improving intestinal motility and promoting intestinal gas clearance (35), while Pilates training may improve bowel function and abdominal discomfort by enhancing parasympathetic activity (36). However, the above mechanisms of action are mostly derived from speculative analyses, and their applicability in the IBS population still requires further validation. It is important to note that the clinical management of IBS usually involves multiple non-pharmacological interventions, and exercise interventions do not exist in isolation. In addition, previous studies have shown that dietary interventions, especially low FODMAP dietary modifications, have clear efficacy in relieving core symptoms such as abdominal pain and bloating in IBS patients (21). In contrast, exercise interventions are more likely to work by improving gut motility, enhancing autonomic nervous system regulation, and optimizing overall functional status. Although the combined effects of exercise and dietary interventions were not analyzed in this study, it is hypothesized based on the available evidence that the two may have a synergistic effect through different but complementary pathways of action, leading to further improvement of IBS symptoms. Given the importance of dietary factors in the management of IBS, this potential confounder may have influenced the results of this study, and future studies may systematically evaluate the combined effects of exercise and dietary interventions under strict control of dietary factors, in order to provide a more comprehensive and evidence-based evidence base for comprehensive intervention strategies in IBS. Although exercise interventions have shown significant effects in improving IBS symptoms, the exact mechanisms are not fully understood. Some studies have proposed that exercise may act by modulating the immune system, reducing inflammation, or improving intestinal barrier function (18, 19), and the available evidence is not sufficient to support immunomodulation as the primary mechanism for IBS symptom improvement. The observed effect is that exercise interventions may work together through multidimensional pathways, including improving psychological status, enhancing autonomic regulation, optimizing gut-brain axis function, improving muscle function, and enhancing overall gut functional status. In addition, interventions such as moderate-intensity aerobic exercise and Pilates may alleviate IBS symptoms through modulation of the gut microbiota, enhancement of gut barrier function, and improvement of autonomic nervous system activity, rather than being limited to direct immunomodulation (18). Therefore, future studies should combine immunological and microbiological indices to distinguish these potential mechanisms more finely, so as to further clarify the biological pathways through which exercise interventions improve IBS symptoms.

It is important to note that some of the exercise interventions in this study presented large effect sizes in the IBS-SSS scale outcome indicators. This phenomenon has also been reported in previous Meta-analyses of non-pharmacological interventions (37). The potential reasons for this may include the following: firstly, the small sample sizes and limited within-group variability of some of the studies predisposed to amplified effect sizes when standardized mean differences (SMDs) or values of change were used for the calculations; secondly, some of the studies reported pre-and post-intervention values of change rather than end-points, which was more likely to result in larger effect sizes in the case of baseline symptomatic are more likely to produce larger effect estimates when they are heavier; and again, the IBS-SSS scale itself is a subjective symptom scale that is more sensitive to behavioral or physical and psychological interventions in short-term interventions, which may also amplify statistical effects. In view of these factors, the present study focuses more on the direction and relative ordering of impact in the interpretation of results rather than the absolute magnitude of a single effect size.

The results of this study indicate a certain degree of statistical heterogeneity among different outcome measures. Analysis of the clinical and methodological characteristics of the included studies suggests that this heterogeneity likely stems primarily from several factors. First, different IBS subtypes (such as IBS-D, IBS-C, and IBS-M) may exhibit variations in pathophysiological features and responsiveness to exercise interventions, thereby contributing to heterogeneous intervention effects. Second, the duration of exercise interventions varied considerably across included studies. Short-term versus long-term interventions may yield differing degrees of symptom improvement, thereby influencing effect size estimates. Additionally, inconsistencies in control group types (e.g., placebo, standard care, or health education) across studies may have impacted estimates of intervention effects. Although subgroup analysis holds potential value in exploring sources of heterogeneity, this study did not conduct quantitative subgroup analyses. This decision was made to avoid unstable results or interpretation bias due to insufficient data, given that some studies did not clearly report stratification by IBS subtype, sample sizes for each subtype were limited, and key information for further stratification was lacking in certain studies. Regarding exercise dosage, while traditional exercise prescriptions typically include elements such as intensity, frequency, and duration, the randomized controlled trials included in this study showed considerable variation in reporting frequency and duration, with incomplete information in some studies. Under these circumstances, attempting to uniformly standardize all dose elements could increase model instability and introduce potential bias. Therefore, this study selected exercise intensity as the primary comparative dimension, while treating exercise frequency and duration as supplementary descriptive indicators.

## 5 Limitations and strengths

The findings of this study should be interpreted with due consideration of their limitations. First, the types of exercise interventions included in the studies varied considerably, encompassing yoga, Pilates, Baduanjin, and moderate-intensity aerobic exercise. Furthermore, some studies employed combined interventions integrating exercise with medication, dietary modifications, or probiotics. This heterogeneity in interventions may have amplified the observed effects to some extent, thereby increasing the inconsistency in effect sizes across studies. Second, control group designs were not uniformly standardized, encompassing conventional drug therapy, dietary interventions, and placebo controls. Differences in control intensity may directly influence effect size estimates. Third, study participants exhibited heterogeneity in IBS subtype composition, age, and disease characteristics. Patients with different subtypes may respond differently to exercise interventions. Combined with inconsistencies in intervention duration, frequency, and exercise dosage, this further increased variability in outcome measures. Fourth, some outcome analyses included a high proportion of small-sample studies, limiting the stability of effect estimates and contributing to overall heterogeneity. Fifth, despite observing substantial inter-study heterogeneity, this review could not conduct subgroup analyses based on IBS subtype, intervention duration, or control group type due to inconsistent reporting of IBS subtypes across included studies, limited sample sizes, and the absence of detailed data for further stratification in some studies. Future studies should more rigorously describe IBS subtypes and intervention characteristics in study design and reporting to support deeper exploration of heterogeneity. Sixth, this study has limitations regarding racial and regional applicability. Due to the limited number of studies involving non-East Asian populations (<3), reliable racial or regional subgroup analyses could not be performed. Consequently, it remains unclear whether the efficacy or mechanisms of exercise interventions in improving IBS symptoms differ across ethnic groups. Although existing studies show consistent improvement trends across regions, their cross-cultural applicability requires validation through future well-designed, adequately powered randomized controlled trials involving multi-ethnic populations. Seventh, this study did not further analyze the impact of dietary interventions on IBS symptoms, despite some control groups employing dietary interventions (e.g., low-FODMAP diet). Future research could explore the combined effects of exercise and diet to more comprehensively evaluate the efficacy of these interventions for IBS.

Due to limitations in the quality and quantity of included studies, different forms of exercise may exert varying effects on these physiological mechanisms. Consequently, future research should conduct large-sample, multicenter high-quality randomized controlled trials to enhance the reliability of evidence; further explore optimal combinations of exercise type, intensity, and frequency; and, by integrating biomarker and gut microbiota analysis, elucidate in depth the mechanisms by which exercise influences IBS. Different exercise intervention approaches demonstrate significant advantages in improving outcomes for IBS patients. The fact that exercise may constitute a safe activity and prove more cost-effective than other treatment strategies for IBS patients supports the future promotion of such interventions in clinical or public health settings.

## 6 Conclusions

This study demonstrates that various exercise interventions can improve clinical symptoms and quality of life in IBS patients across different outcome measures. Results from the network meta-analysis indicate that running and Baduanjin exhibit higher relative effectiveness in improving the IBS-SSS and IBS-QOL total scale, while moderate-intensity aerobic exercise shows greater efficacy in alleviating anxiety symptoms. Different exercise modalities exhibit “functional specificity” across outcome dimensions, with no single exercise demonstrating absolute superiority across all measures. As a safe, low-cost non-pharmacological intervention, exercise may hold clinical value in the comprehensive management of IBS. Existing research suggests exercise intervention may alleviate IBS symptoms through multiple pathways, including regulating autonomic nervous function, improving intestinal motility, enhancing muscle function, and optimizing intestinal barrier function. However, these mechanisms are largely inferred indirectly, and direct evidence supporting immune modulation as the primary mechanism for exercise-induced symptom improvement remains lacking. Future studies should systematically validate the potential biological mechanisms of exercise intervention by integrating immunology, biomarkers, and gut microbiome indicators. Due to insufficient literature on non-East Asian populations (<3 studies), this research could not perform reliable racial subgroup analyses. Consequently, the applicability of these findings across different ethnicities remains unclear. Future studies should include patients from diverse geographic regions and ethnic groups to further validate the efficacy of exercise interventions across racial populations.

## Data Availability

The original contributions presented in the study are included in the article/supplementary material. Further inquiries can be directed to the corresponding author/s.
